# Lineage Tracing of Pf4-Cre Marks Hematopoietic Stem Cells and Their Progeny

**DOI:** 10.1371/journal.pone.0051361

**Published:** 2012-12-27

**Authors:** Simon D. J. Calaminus, Amelie Guitart, Amy Sinclair, Hannah Schachtner, Steve P. Watson, Tessa L. Holyoake, Kamil R. Kranc, Laura M. Machesky

**Affiliations:** 1 Beatson Institute for Cancer Research, University of Glasgow, Glasgow, United Kingdom; 2 Paul O’Gorman Leukaemia Research Centre, Institute of Cancer Sciences, University of Glasgow, Glasgow, United Kingdom; 3 Centre for Cardiovascular Sciences, Institute for Biomedical Research, University of Birmingham, Birmingham, United Kingdom; University of Leuven, Belgium

## Abstract

The development of a megakaryocyte lineage specific Cre deleter, using the Pf4 (CXCL4) promoter (Pf4-Cre), was a significant step forward in the specific analysis of platelet and megakaryocyte cell biology. However, in the present study we have employed a sensitive reporter-based approach to demonstrate that Pf4-Cre also recombines in a significant proportion of both fetal liver and bone marrow hematopoietic stem cells (HSCs), including the most primitive fraction containing the long-term repopulating HSCs. Consequently, we demonstrate that Pf4-Cre activity is not megakaryocyte lineage-specific but extends to other myeloid and lymphoid lineages at significant levels between 15–60%. Finally, we show for the first time that Pf4 transcripts are present in adult HSCs and primitive hematopoietic progenitor cells. These results have fundamental implications for the use of the Pf4-Cre mouse model and for our understanding of a possible role for Pf4 in the development of the hematopoietic lineage.

## Introduction

Cre-loxP technology allows the deletion or expression of genes to be regulated temporally and spatially in animals [1], [2], [3], [4]. The nature of a specific promoter used to drive Cre recombinase expression determines the tissue specificity and timing of expression. Several tissue-specific Cre deleter strains have been developed in order to study the effects of conditional gene expression or deletion in a specific tissue or organ system [2], [5], [6]. The recent arrival of a megakaryocyte specific Cre driven by the platelet factor 4 promoter (Pf4-Cre) [7] has significantly impacted on platelet and megakaryocyte studies [8], [9], [10]. This mouse model contributed towards the confirmation of the role of platelets in the separation of the lymphatic and vasculature systems [8], [11]. However, there are reports that endogenous Pf4 is expressed in cells outside of the megakaryocyte lineage, for example osteoclasts and monocytes [12], [13], suggesting that the Pf4 promoter may not be specific to the platelet-megakaryocyte lineage.

Here we performed lineage-tracing studies revealing that Pf4-Cre marks a portion of fetal liver and bone marrow hematopoietic stem cells (HSCs) and their downstream progeny. Consistent with this, we show that Pf4 transcripts are present in HSCs and primitive hematopoietic cells. Taken together, these results provide genetic evidence that Pf4-Cre does not recombine solely in the megakaryocyte lineage but in addition in a large proportion of stem and progenitor cells and all their progeny. Therefore, our results imply that hematopoietic phenotypes of conditional mutations generated using the Pf4-Cre deleter must be interpreted with caution. These observations additionally indicate a possible role for PF4 in haematopoietic stem or progenitor function, which merits future investigation.

## Materials and Methods

### Transgenic Mice

All experiments were carried out according to UK Home Office regulations. We crossed Pf4-Cre transgenic mice [7] with mice containing a conditional tandem dimer RFP (tdRFP) construct under the control of the Rosa26 promoter [14]. Rosa26-tdRFP^−^;Pf4-Cre^+^ and Rosa26-tdRFP^+^;Pf4-Cre^−^ mice were used as controls. The Rosa26-tdRFP^+^;Pf4-Cre^+^ mice were heterozygous at the Rosa26-tdRFP locus.

### Flow Cytometry

Cells were stained as previously described [15], [16]. Platelets and megakaryocytes were isolated from the peripheral blood and the bone marrow according to standard procedures [17], [18]. FACS analysis was performed using the FACSCanto Flow Cytometer and cell isolation was performed using the FACSAria II cell sorter (Becton Dickinson, UK) and analysed using FlowJo software (Tree Star Incorporation, USA).

### qPCR

For qPCR, whole bone marrow was stained with antibodies against a lineage cocktail, Sca-1, c-kit and isolated based on CD150 and CD48 staining. Single cells were sorted using the cells direct one-step qRT-PCR kit (Life Technologies, USA) and gene expression carried out on a Fluidigm chip (Fluidigm, USA) with Taqman probes (Life Technologies, USA). Beta-2-Microglobulin was used as the housekeeping control. The Lin^−^Sca-1^+^c-kit^+^ (LSK) CD150^+^CD48^−^ HSC population (defined as the most primitive HSC fraction or Long-term HSC (LT-HSC)) was used as a calibrator and set to the value of 1. The fold change of Pf4 in all other populations was in comparison to LT-HSC population. The data were calculated using the Delta Delta CT method for qPCR analysis [15], [16].

### Histology

Mouse femur and tibia were removed and placed either in ice-cold formalin for overnight fixation (quick fix) or room-temperature for long fixation, and then processed into paraffin blocks. Tissue sections were then cut, and processed for RFP immunohistochemistry. Slides were de-waxed for 2×10 min washes in xylene, and then hydrated by washing in decreasing concentrations of alcohol 100%, 95%, and 70% for 3 mins, followed by a final wash in tap water. The antigen is retrieved by boiling slides in citrate buffer pH6 for 4 min in a microwave. The slides were cooled, before rinsing in dH_2_O, and blocked with 3% hydrogen peroxide for 15 min, followed by 3 washes in TBS-T/0.05%Tween. The slides were blocked for 1 hr with 5% goat serum, and incubated overnight at 4°C with 1/200 RFP antibody (Abcam, Cambridge, UK). Slides were then washed before addition of secondary antibody following the Envision HRP kit protocol (Dako, Cambridge, UK). The slides were visualized for 2 min before termination with dH_2_O. The slides were counterstained with Meyers Haematoxylin before mounting.

## Results and Discussion

**Figure 1:**
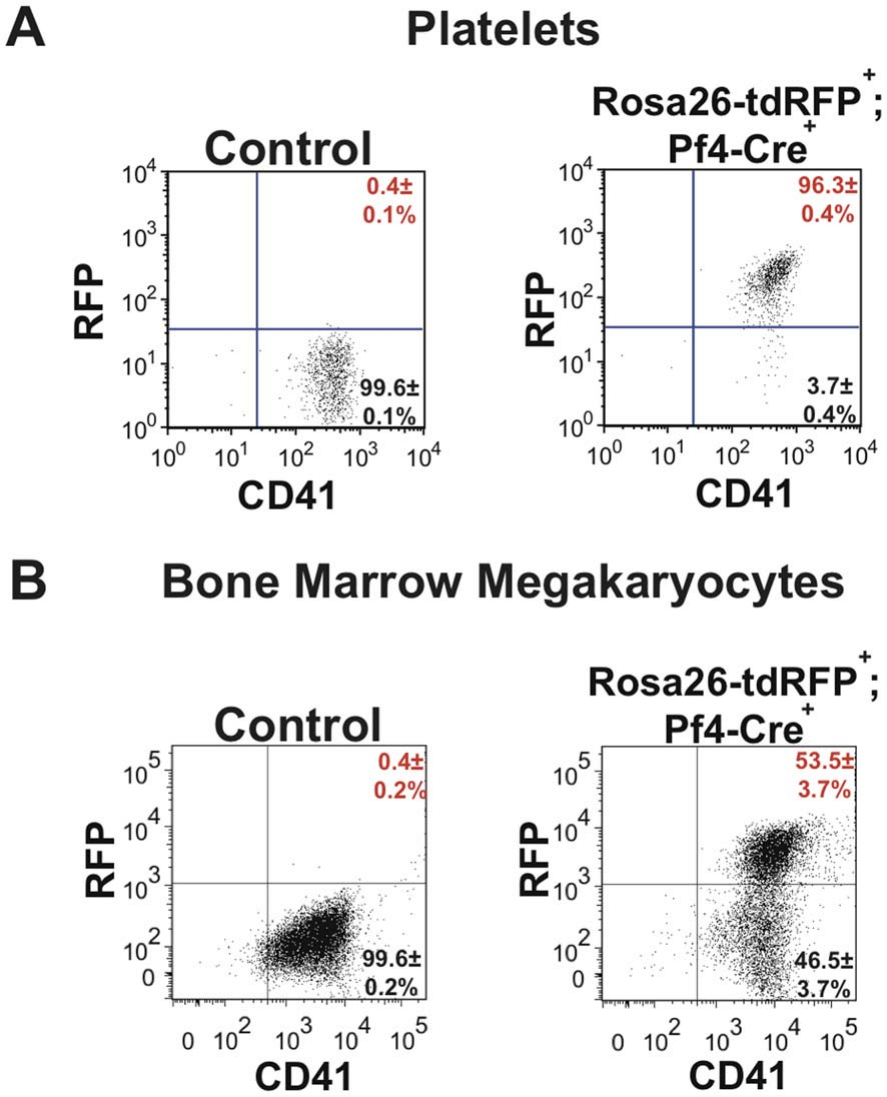
Pf4-Cre efficiently excises floxed sequences in megakaryocytes and platelets. (**A**) Platelets from both Rosa26-tdRFP^+^;Pf4-Cre^−^ litter matched control and Rosa26-tdRFP^+^;Pf4-Cre^+^ mice were isolated from whole blood before being stained with anti-CD41-FITC antibody. The level of RFP expression in the platelet (CD41^+^) population was identified. (**B**) Bone marrow megakaryocytes were isolated before being stained with anti-CD41-FITC antibody. Megakaryocytes were identified using FSC vs SSC gating and the levels of RFP expression in the megakaryocyte population was determined. Dot plots are representative figures of three independent experiments with the mean±standard error of the mean (SEM).

To perform lineage tracing of Pf4-Cre we crossed Pf4-Cre transgenic mice with mice containing a conditional loxP flanked NEO-STOP-tdRFP under the control of the Rosa26 promoter [14]. First we confirmed efficient Pf4-Cre-mediated recombination in platelets and megakaryocytes. As expected almost all platelets from Rosa26-tdRFP^+^;Pf4-Cre^+^ adult mice expressed RFP (Fig. 1A). The analysis of the megakaryocyte population isolated from the bone marrow of Rosa26-tdRFP^+^;Pf4-Cre^+^ mice revealed that over 53.5±3.7% of cells expressed RFP (Fig. 1B). This result is lower than that seen by Tiedt et al but this is likely to be due to the presence of immature megakaryocytes which have not yet undergone recombination and also those that will need time to switch on RFP expression. However, our data indicate that all mature platelet producing megakaryocytic have undergone recombination as expected. Indeed even Tiedt et al identified that in the megakaryocyte population integrin β1 was not completely deleted in integrinβ1^flox/flox^Pf4-Cre^+^ indicating that possibly not all purified megakaryocytes have yet undergone recombination.

**Figure 2:**
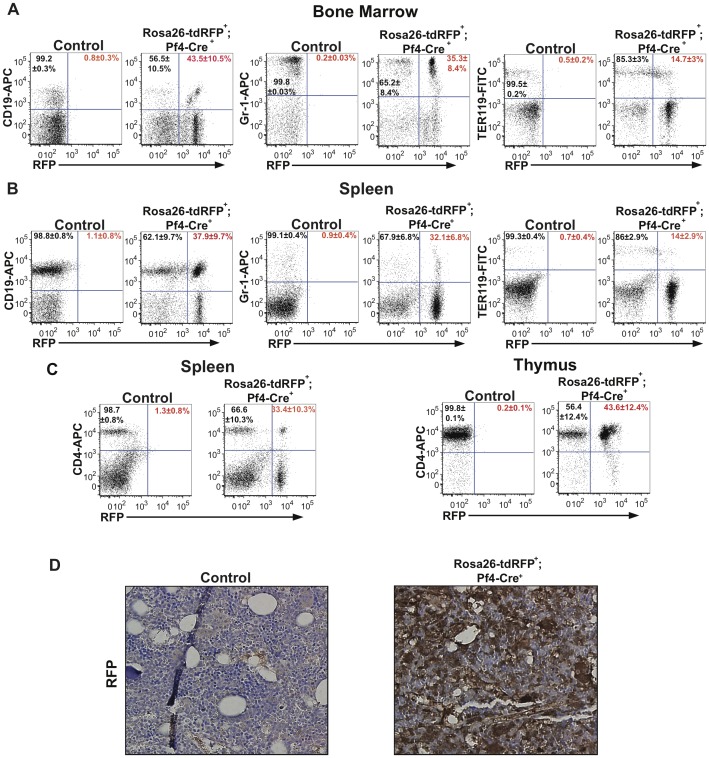
Pf4-Cre activates RFP expression in all hematopoietic lineages. (**A**) Frequencies of RFP-expressing B-lymphoid (CD19^+^), myeloid (Gr-1^+^), and erythroid (Ter119^+^) cells in the bone marrow obtained from adult Rosa26-tdRFP^+^;Pf4-Cre^+^ mice and Rosa26-tdRFP^+^;Pf4-Cre^−^ litter matched controls. (**B**) Frequencies of RFP-expressing B-lymphoid (CD19^+^), myeloid (Gr-1^+^), and erythroid (Ter119^+^) cells in the spleen from adult Rosa26-tdRFP^+^;Pf4-Cre^+^ mice and Rosa26-tdRFP^+^;Pf4-Cre^−^ litter matched controls. (**C**) Frequencies of RFP-expressing CD4^+^ T cells in the spleen and thymus of adult Rosa26-tdRFP^+^;Pf4-Cre^+^ mice and Rosa26-tdRFP^+^;Pf4-Cre^−^ litter matched controls. Dot plots shown in Figure 1A–C are representative figures of 3–5 independent experiments with values shown as an average±SEM from 3–5 independent experiments. % of RFP^+^ cells is indicated in red and % of RFP^−^ in black. (**D**) Bone marrow from Rosa26-tdRFP^+^;Pf4-Cre^+^ and litter matched controls was fixed, and histologically stained for RFP. Images are representative of 2 independent experiments.

Next, we determined if Pf4-Cre was activated outside the megakaryocyte/platelet lineage. The analysis of Rosa26-tdRFP^+^;Pf4-Cre^+^ adult mice (3–9 months of age) revealed RFP expression in 43.5±10.5% mature B-lymphoid (CD19^+^), 35.3±8.4% myeloid (Gr-1^+^) and 14.7±3% erythroid (Ter119^+^) bone marrow lineages (Fig. 2A) in comparison to controls. Further analysis of the Rosa26-tdRFP^+^;Pf4-Cre^+^ spleens indicated 37.9±9.7% mature B cells, 32.1±6.8% myeloid, 14±2.9% erythroid and 33.4±10.3% T cells (CD4^+^ cells) with RFP expression, whilst in the thymus 43.6±12.4% T cells had RFP expression (Fig. 2B&C). This expression of RFP outside the megakaryocyte lineage was confirmed by immunostaining for RFP expression in bone marrow histology samples (Fig. 2D). Our flow cytometry analysis indicates RFP^+^ populations in all mature blood lineages, demonstrating that Pf4-Cre activity is not restricted to the megakaryocytic lineage.

**Figure 3:**
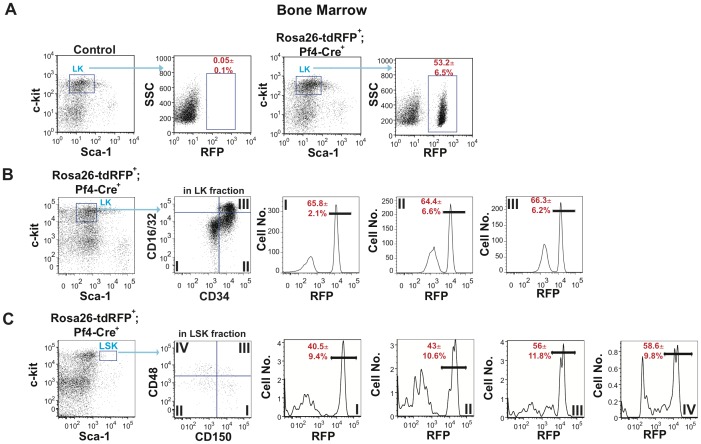
Pf4-Cre genetically marks the adult stem cell and early progenitor cell compartment. Bone marrow from Rosa26-tdRFP^+^;Pf4-Cre^+^ and litter matched control mice was isolated and (**A**) the Sca-1^−^c-kit^+^ cells identified in the Lin^−^ cell compartment. LK compartment was subfractionated using side scatter (SSC) and RFP expression to identify the total number of RFP^−^ (black) and RFP^+^ (red) cells. (**B**) The LK population was subfractionated using CD34 and CD16/32 to identify RFP^+^ (red) cells in the megakaryocyte-erythrocyte progenitor (MEP) (Compartment I), common myeloid progenitor (CMP) (Compartment II) and granulocyte-macrophage progenitor (GMP) (Compartment III) cell populations. (**C**) RFP expression in HSCs and primitive progenitors in the LSK compartment. The LSK cell compartment was subfractionated using CD48 and CD150 to identify the frequencies of RFP^+^ (red) cells in LSK CD150^+^CD48^−^ HSC (Compartment I), LSK CD150^−^CD48^−^ (Compartment II), LSK CD150^+^CD48^+^ (Compartment III) and LSK CD150^−^CD48^+^ (Compartment IV) compartments. Dot plots and histograms are representative figures of three independent experiments with the mean±SEM from three independent experiments.

The multilineage expression of RFP in Rosa26-tdRFP^+^;Pf4-Cre^+^ mice suggested that Pf4-Cre could be active in either primitive progenitors or HSCs. Analysis of the Lin^−^c-kit^+^ (LK) compartment, which contains the common myeloid progenitor (CMP), granulocyte myeloid progenitor (GMP) and megakaryocyte-erythrocyte progenitor (MEP) cell populations, contained just over 50% of RFP expressing cells (Fig. 3A). The frequency of RFP^+^ cells in the MEP, CMP and GMP populations was 65.8±2.1%, 64.4±6.6% and 66.3±6.2% respectively (Fig. 3B). Further analyses of Rosa26-tdRFP^+^;Pf4-Cre^+^ mice showed significant RFP expression in the LSK compartment, which contains the LT-HSCs, short-term HSCs (ST-HSCs), and lymphoid primed multipotent progenitors (LMPPs). On average 54.8±6.4% of cells in the LSK compartment expressed RFP (data not shown). To identify the most primitive hematopoietic cell type that expresses Pf4, we subfractionated the LSK compartment of Rosa26-tdRFP^+^;Pf4-Cre^+^ mice using CD150 and CD48 staining. Our data indicated that 40.5±9.4% of LSK CD150^+^CD48^−^ HSCs (LT-HSCs) expressed RFP (Fig. 3C). Furthermore, the downstream progeny of LT-HSCs, ST-HSCs (CD150^−^CD48^−^:Compartment II), MPP1 (CD150^+^CD48^+^:Compartment III), and MPP2 (CD150^−^CD48^+^:Compartment IV) all expressed RFP (Fig. 3C). Reanalysis of the LSK and LK compartments with a CD41 gate to remove possible platelet contamination identified 55.6±2.8% of CD41^−^ cells were RFP^+^ in the LSK compartment whilst in the LK compartment 55.6±2.1% of CD41^−^ cells were RFP^+^ (data not shown). Therefore, RFP expression in the stem cell and progenitor compartments is not due to contamination from a platelet or megakaryocyte population. These immunophenotypic analyses demonstrated that Pf4-Cre exhibits its activity in the most primitive compartment of the adult hematopoietic differentiation hierarchy.

**Figure 4:**
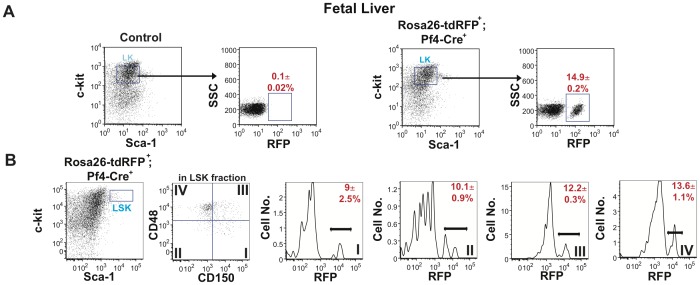
Primitive fetal liver cells from Rosa26-tdRFP^+^;Pf4-Cre^+^ mice express RFP. Fetal liver from day E14.5 Rosa26-tdRFP^+^;Pf4-Cre^+^ and litter matched controls was isolated and (**A**) the Sca-1^−^c-kit^+^ cells identified in the Lin^−^ cell compartment. LK compartment was subfractionated using side scatter (SSC) and RFP expression to identify the total number of RFP^−^ (black) and RFP^+^ (red) cells. Dot plots are representative figures of three independent experiments with the average±SEM from three independent experiments. (**B**) RFP expression in HSCs and primitive progenitors within the LSK compartment. The LSK cell compartment was subfractionated using CD48 and CD150 to identify the frequencies of RFP^+^ (red) in LSK CD150^+^CD48^−^ HSC (Compartment I), LSK CD150^−^CD48^−^ (Compartment II), LSK CD150^+^CD48^+^ (Compartment III) and LSK CD150^−^CD48^+^ (Compartment IV) compartments. Dot plots and histograms are representative figures of three independent experiments with the mean±SEM from three independent experiments.

Next, we determined whether Pf4-Cre also marks fetal HSCs. Immunophenotypic analyses of fetal liver from 15.5 dpc Rosa26-tdRFP^+^;Pf4-Cre^+^ and control embryos revealed there was also a significant population of RFP^+^ cells in the LK compartment (Fig. 4A). This RFP expression was replicated in the LSK compartment with RFP expression in 12.2±0.6% of LSK cells (data not shown). Furthermore, 9±2.5% of LSK CD150^+^CD48^−^ HSCs expressed RFP (Fig. 4B). As the LT-HSCs became ST-HSCs and LMPPs, there was an increase in RFP expression in these compartments (Fig. 4B). These data indicate that the Pf4-Cre transgene is activated in fetal liver HSCs during embryonic development.

**Figure 5:**
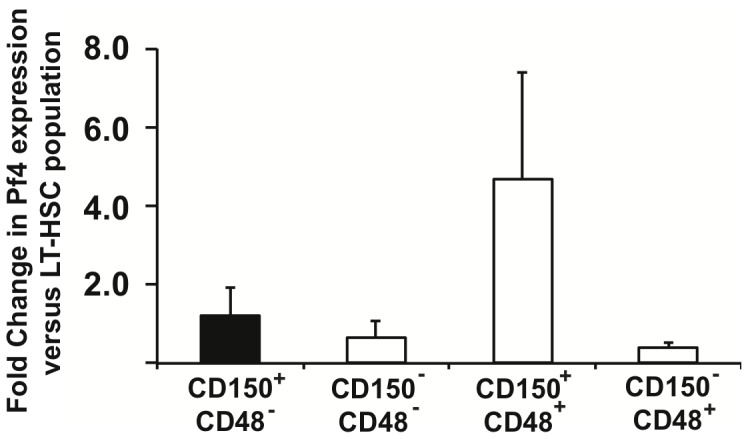
Pf4 gene expression is identified in Stem and primitive progenitor cell populations. Pf4 expression in LSK CD150^+^CD48^−^ HSCs, LSK CD150^−^CD48^−^, LSK CD150^+^CD48^+^, and LSK CD150^−^CD48^+^ cells sorted from bone marrow of control mice. Relative Pf4 expression levels were calculated with the LT-HSC fraction set to the value of 1 (see methods). Results are mean±SD from three independent experiments.

The presence of RFP in HSCs and their progeny raised a question of whether the endogenous Pf4 gene might also be expressed in the most primitive hematopoietic cells. To address this, we determined the expression of endogenous Pf4 in LSK CD150^+^CD48^−^ HSCs (LT-HSCs) isolated from genetically normal mice, and their immediate downstream progeny (LSK CD150^−^CD48^−^, LSK CD150^+^CD48^+^and LSK CD150^−^CD48^+^ cells) using qPCR. These analyses revealed that endogenous Pf4 transcripts are present in all tested populations of the LSK compartment (Fig. 5). The presence of endogenous Pf4 mRNA in HSCs and primitive progenitor cells reflects the transcriptional activity of the Pf4 promoter in these populations and, at least in part, explains the multilineage expression of RFP in Rosa26-tdRFP^+^;Pf4-Cre^+^ mice.

We demonstrate that Pf4-Cre is activated not only in the megakaryocyte lineage but additionally in approximately 50% of stem and primitive progenitor cell compartments. This leads to expression of the RFP reporter in all hematopoietic lineages of the Rosa26-tdRFP^+^;Pf4-Cre^+^ mice. In agreement with our data, Chagraoui et al used semi-quantitative PCR to identify between 30–50% recombination in CMP, GMP and MEP progenitors in SCL^fx/fx^Pf4iCre^+^ mice [19]. However, Tiedt et al [7] and Bertozzi et al [8] have inferred the specificity of the Pf4-Cre to the megakaryocyte lineage through the use of histology and immunostaining. We believe there are technical reasons why these results differ to our results. Histology, in our hands showed variable staining, depending on whether a long fixation or quick fixation protocol was used. Secondly, the mouse model we have used contains a tandem RFP. This reporter has increased fluorescence intensity in comparison to other reporter mice [14], and therefore may be more sensitive for identifying expression in other compartments than either the LacZ or YFP reporters.

Our data indicate that the Pf4 expression is not restricted to the megakaryocytic/platelet lineage and is also present in HSCs and their primitive progeny. This observation, taken together with the expression of another megakaryocytic/platelet lineage-associated gene VWf [20] in self-renewing HSCs fits the model in which HSCs express individual lineage-affiliated genes [21]. The functional significance of the expression of megakaryocytic/platelet lineage-associated genes in HSCs remains an open question meriting future investigations.

Finally, our results provide genetic evidence indicating that hematopoietic phenotypes of conditional mutations generated using the Pf4-Cre deleter must be interpreted in light of Pf4-Cre activation outside the megakaryocyte lineage. It is fundamental to understand the effect of this leakiness outside the megakaryocyte lineage in each conditional mouse used to be able to fully interpret results obtained from the use of the Pf4-Cre mouse model system.
