# Correction: Lineage Tracing of Pf4-Cre Marks Hematopoietic Stem Cells and Their Progeny

**DOI:** 10.1371/annotation/4c610387-c7f0-4330-b772-30ed52a07547

**Published:** 2013-05-31

**Authors:** Simon D. J. Calaminus, Amelie V. Guitart, Amy Sinclair, Hannah Schachtner, Steve P. Watson, Tessa L. Holyoake, Kamil R. Kranc, Laura M. Machesky

The correct name of the second author is: Amelie V. Guitart

The correct citation is: Calaminus SDJ, Guitart AV, Sinclair A, Schachtner H, Watson SP, et al. (2012) Lineage Tracing of Pf4-Cre Marks Hematopoietic Stem Cells and Their Progeny. PLoS ONE 7(12): e51361. doi:10.1371/journal.pone.0051361 

